# Supplementary material for: Health awareness and the transition towards clean cooking fuels: Evidence from Rajasthan
Source: PLoS One. 2020 Apr 29;15(4):e0231931. doi: 10.1371/journal.pone.0231931 (PMC7190100; doi:10.1371/journal.pone.0231931)
Supplement: S1 Appendix — (PDF) [file pone.0231931.s001.pdf]

Mathematical derivations for the theoretical model

In this paper, we proposed an illustrative model based on the Cobb-Douglas utility function:

$$U(g,x) = g^{\theta} x^{1-\theta} \tag{1}$$

where g is the cooking gas LPG, x is a composite good that includes traditional biomass and other goods, and $\theta \in [0,1]$ is an indicator for the preference for LPG as compared to the composite good.

For an income B and prices p_g and p_x, the budget constraint is:

$$gp_g + xp_x \leq B \tag{2}$$

Maximizing (1) subject to (2) yields the Marshallian demand function for LPG:

$$g^*(p_g) = \frac{B}{p_g} \cdot \theta \tag{3}$$

Inverting this function we obtain the price a household is willing to pay for this quantity of LPG:

$$p_g(g^*) = \frac{B}{g^*} \cdot \theta. \tag{4}$$

Predictions related to WTP

Imagine we request the household to increase its consumption from g^* to $\bar{g} = 2g^*$ as we are interested in WTP for regular rather than very sporadic users. Assume that such a doubling of LPG consumption is feasible within the budget constraint (Assumption 1, see section Assumptions). To make this situation again optimal for the household, the new price must be 50% lower than the initial price:

$$p_g(\bar{g}) = \frac{B}{\bar{g}} \cdot \theta = \frac{1}{2} \cdot p_g(g^*). \tag{5}$$

While this exact relationship is directly related to the restrictive assumption underlying the Cobb-Douglas utility function that the price-elasticity of demand is equal to 1, even otherwise, we would clearly expect a reduction in WTP with an increase in the requested amount to be consumed.

More interesting in the context of our study, however, is the question to what extent health messaging can compensate some of this reduction in WTP. What is the change in WTP if we increase the decision maker's knowledge about the adverse health effects of cooking with traditional biomass?

Let us consider the preference for LPG θ as a linear function of health knowledge $h \in [0,1]$:

$$\theta = \bar{\theta} + h \cdot \gamma \tag{6}$$

where $\bar{\theta} \in [0,1]$ is basic preference (e.g., due to the convenience and time savings associated with LPG) and $\gamma \in [0,1]$ is a factor reflecting the salience of health information, notably due to gender.

We can then rewrite $p_g(\bar{g})$ as:

$$p_g(\bar{g}) = \frac{B}{\bar{g}} \cdot (\bar{\theta} + h \cdot \gamma) \tag{7}$$

The expected effect of health messaging on WTP is then given by:

$$\frac{\partial p_g}{\partial h} = \frac{B}{\bar{g}} \cdot \gamma > 0 \tag{8}$$

We are further interested to see if the impact of health messaging is affected by differences related to gender reflected by differences in the salience of the health information. To see this we need to take the cross-derivative with respect to h and γ. To do so, note that factors such as gender already influence the initial value of g^* and thus also $\bar{g}$. More formally, we can write:

$$\bar{g} = 2g^*(p_m, \bar{\bar{\theta}}) = 2\frac{B}{p_m} \cdot (\bar{\theta} + \bar{h} \cdot \gamma) \tag{9}$$

where p_m is the original market price and $\bar{\bar{\theta}} = \bar{\theta} + \bar{h} \cdot \gamma$ the initial preference for LPG. In other words, $\bar{g}$ is fixed as the double of the optimal consumption at the general market price and the initial preference for LPG $\bar{\bar{\theta}}$ that is based on the initial health knowledge and salience. We keep h fixed at this initial level $\bar{h}$ as its change due to the treatment does not influence g^*. In contrast, a greater salience of such health knowledge γ already influences the initial g^*. Hence, $\bar{g}$ needs to be considered as a function of γ but not of h when we take the derivatives. We assume that the treatment itself does not affect γ (Assumption 2, see section Assumptions), which is obvious if we think of it as reflecting the gender of the decision maker.

Inserting (9) in (8) and taking the derivative with respect to γ, we obtain:

$$\frac{\partial^2 p_g}{\partial h \partial \gamma} = \frac{\bar{\theta} p_m}{2(\bar{\theta} + \bar{h}\gamma)^2} > 0 \tag{10}$$

Predictions related to the propensity of voucher use

The propensity to use the voucher can be expressed as the difference in utility ΔU between a situation in which the voucher is used U_1 and a situation in which it is not used U_0. Taking into account the conditions for voucher use, namely doubling initial consumption and the discounted offer price p_d, we can specify U_1 as

$$U_1 = \bar{g}^{\theta} x^{1-\theta} = \bar{g}^{\theta} (B - \bar{g} p_d)^{1-\theta} \tag{11}$$

In contrast, the utility when the voucher is not used U_0 simply corresponds to Eq (1) evaluated at the optimal level of consumption given the market price p_m, without any discount but with the possibility to freely adjust all quantities to changes in θ:

$$U_0 = g^{*\theta} x^{*1-\theta} \tag{12}$$

ΔU can thus be rewritten as

$$\Delta U = U_1 - U_0 = \bar{g}^{\theta} (B - \bar{g} p_d)^{1-\theta} - g^{*\theta} x^{*1-\theta}. \tag{13}$$

To facilitate the computation of the derivatives we simplify Eq (13) through a monotonous transformation using logs. This transformation will leave the sign of the derivatives unchanged.

$$
\begin{aligned}
\Delta u &= \ln U_1 - \ln U_0 \\
&= (1-\theta)\ln\frac{B - 2g^*(p_m,\bar{\bar{\theta}})p_d}{B - g^*(p_m,\theta)p_m} + \theta \ln \frac{2g^*(p_m,\bar{\bar{\theta}})}{g^*(p_m,\theta)} \\
&= (1-\theta)\ln(1 - 2\bar{\bar{\theta}}\frac{p_d}{p_m}) - (1-\theta)\ln(1-\theta) + \theta\ln(2\bar{\bar{\theta}}) - \theta\ln\theta
\end{aligned} \tag{14}
$$

Replacing θ by (6) and taking the derivative with respect to h yields:

$$
\frac{\partial \Delta u}{\partial h} = -\gamma \ln\left(\frac{1 - 2\bar{\bar{\theta}}\frac{p_d}{p_m}}{2\bar{\bar{\theta}}}\right) + \gamma \ln\left(\frac{1-\theta}{\theta}\right) > 0 \tag{15}
$$

Note that this computation is again based on Assumption 1 (a doubling of consumption is feasible within the budget constraint), or else, we would take the log of a negative quantity in the first term. A further relevant assumption is that the requirement to double LPG consumption in order to use the voucher is a binding constraint (Assumption 3, see section Assumptions). For more extreme preferences for LPG, the model would suggest that the household would forego the voucher in order to be able to consume more LPG. This situation is irrelevant in practice, as the voucher can also be used any time before the deadline, and hence there is no constraint on the maximum use of LPG. For reasons of simplification, the model has not been designed to cover these obvious cases where the health treatment is extremely effective. Finally, remember that $0 < \frac{p_d}{p_m} \leq 1$ since p_d is the discounted price while p_m is the market price. Considering all these arguments, we obtain the sign of the derivative.

We now examine how the impact of h on Δu varies for different levels of salience of health information. We use (15) evaluated at the initial preferences for LPG $\theta = \bar{\bar{\theta}}$. Considering that $\bar{\bar{\theta}} = \bar{\theta} + \bar{h} \cdot \gamma$ we can take the derivative of $\frac{\partial \Delta u}{\partial h}$ with respect to γ to obtain the cross-derivative:

$$
\frac{\partial^2 \Delta u}{\partial \mathrm{h} \partial \gamma} = \ln\left(\frac{2(1-\bar{\bar{\theta}})}{1 - 2\bar{\bar{\theta}}\frac{p_d}{p_m}}\right) + \gamma \frac{h \cdot (2\frac{p_d}{p_m} - 1)}{(1 - 2\bar{\bar{\theta}}\frac{p_d}{p_m})(1-\bar{\bar{\theta}})} > 0 \tag{16}
$$

This inequality holds under exactly the same conditions as the inequality in (15).

Before concluding this analysis, let us further examine the reaction of Δu to a change in the discounted offer price p_d. Since this price can be obtained only when the household effectively uses the voucher, a lower p_d makes voucher use more attractive:

$$
\frac{\partial \Delta u}{\partial p_d} = -\frac{(1-\theta)2\theta}{(1-2\theta)p_m} < 0 \tag{17}
$$

This inequality only requires Assumption 1 (see section Assumptions). The negative relationship between WTP and the required consumption is thus also reflected in the lower propensity of voucher use (implying the doubling of consumption) for higher p_d.

Finally, note that—as opposed to WTP—the propensity of voucher use is unrelated to the budget B, since it enters in the same way in both U_1 and U_0 and hence cancels out:

$$
\frac{\partial \Delta u}{\partial B} = 0 \tag{18}
$$

Assumptions This section provides an overview of the three main assumptions referred to above:

1. Doubling LPG consumption (as imposed in the experiment) is theoretically possible, i.e., the consumption of other goods does not fall below 0 for all possible prices $p_d \in [0.5p_m, p_m]$ and θ. Formally,

$$B - 2g^*(p_m, \theta) \cdot p_d > 0, \forall p_d, \forall \theta$$

   Using Eq (3) this further implies: $1 - 2\theta > 0, \forall \theta$.

2. The treatment $d \in [0, 1]$ does not alter γ directly, i.e., health messaging only affects health knowledge h, but not the salience of this knowledge:

$$\frac{\partial \gamma}{\partial d} = 0$$

   This is certainly true for the main variable we think of in this context, namely gender, but also smoke exposure more broadly, which cannot change immediately, i.e., prior to the household's reactions with respect to consumption or stated WTP that we are assessing here.

3. The preference increase for LPG as a result of the intervention is not so strong that the household would want to increase its LPG use by more than 100%. This implies that the requirement we impose on the household to *at least double* its LPG consumption can be treated in the model as a requirement to *double* consumption:

$$\bar{g} = \max\{2g^*(p_m, \bar{\bar{\theta}}), g^*(p_d, \theta)\} = 2g^*(p_m, \bar{\bar{\theta}}) \Rightarrow 2\bar{\bar{\theta}} > \theta$$

   The alternative case is of course possible, but including this option into the model would make the model more complex, while not changing anything substantially. This is because households willing to consume more than $2g^*(p_m, \bar{\bar{\theta}})$ will have an even higher propensity to use the vouchers.
