# Supplementary material for: Health awareness and the transition towards clean cooking fuels: Evidence from Rajasthan
Source: PLoS One. 2020 Apr 29;15(4):e0231931. doi: 10.1371/journal.pone.0231931 (PMC7190100; doi:10.1371/journal.pone.0231931)

Power calculation and sampling protocol

We determined our sample size based on the aim to detect an additional WTP for LPG associated with the intervention of 12 INR or larger. 12 INR corresponded to 2.5% of the regulated market rate for a standard size LPG cylinder when the field study started (= 480 INR). Assuming that the pooled standard deviation of WTP would be 60 INR (based on a pilot among 21 households), the price difference of 12 INR corresponded to a between-groups effect size of d=.2 (small). To obtain statistical power at the recommended 0.8 level with alpha set at 0.05 for a two-tailed test, a sample of 393 would be required. However, if the variance is higher, the required sample size increases substantially. We hence aimed at 500 usable observations for the experiment. Adding 10% to account for different kinds of data problems which may arise resulted in 550 planned interviews. We hence sampled 55 villages in Bikaner district with probability proportional to population size. Fig 1 shows this district, in the state of Rajasthan. The protocol in Fig 2 describes the sampling procedure in detail.

Fig 1. Study area of Bikaner district, Rajasthan, India.

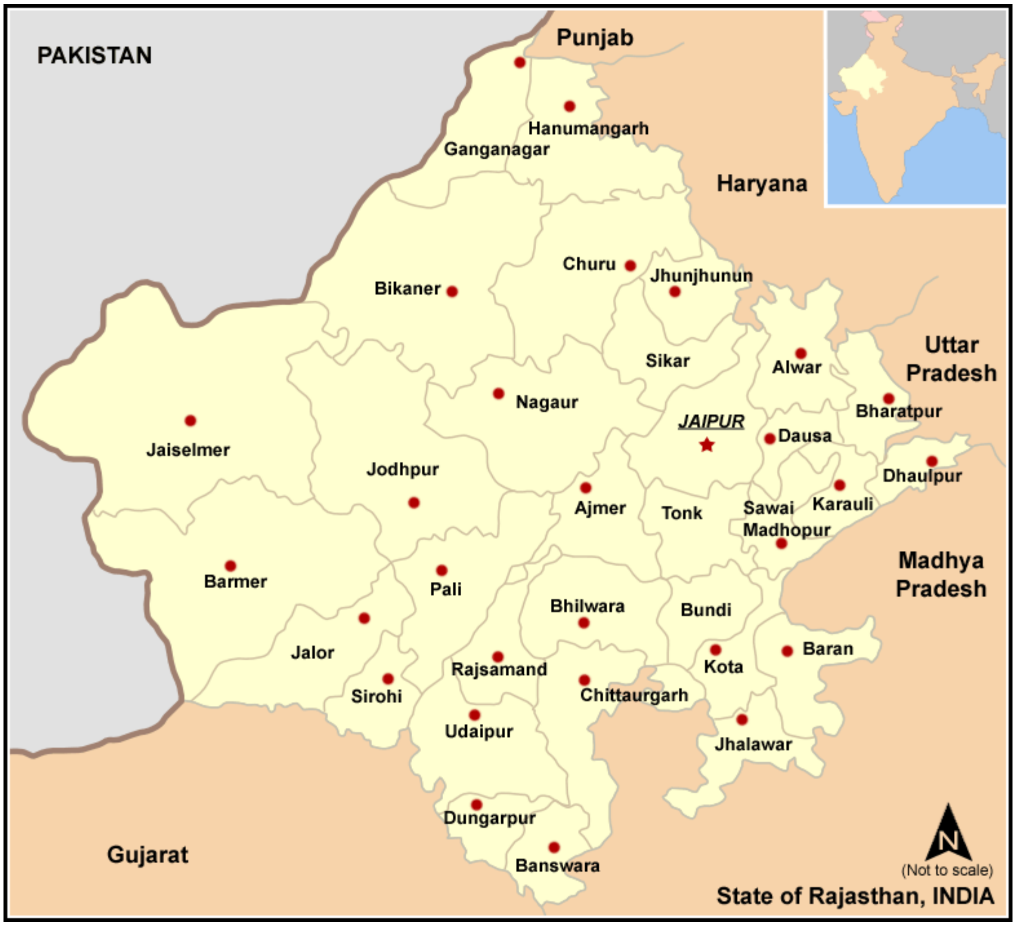

Source: By Miljoshi, available on wikimedia commons under the CC BY-SA 3.0 license.

Fig 2. Sampling protocol.

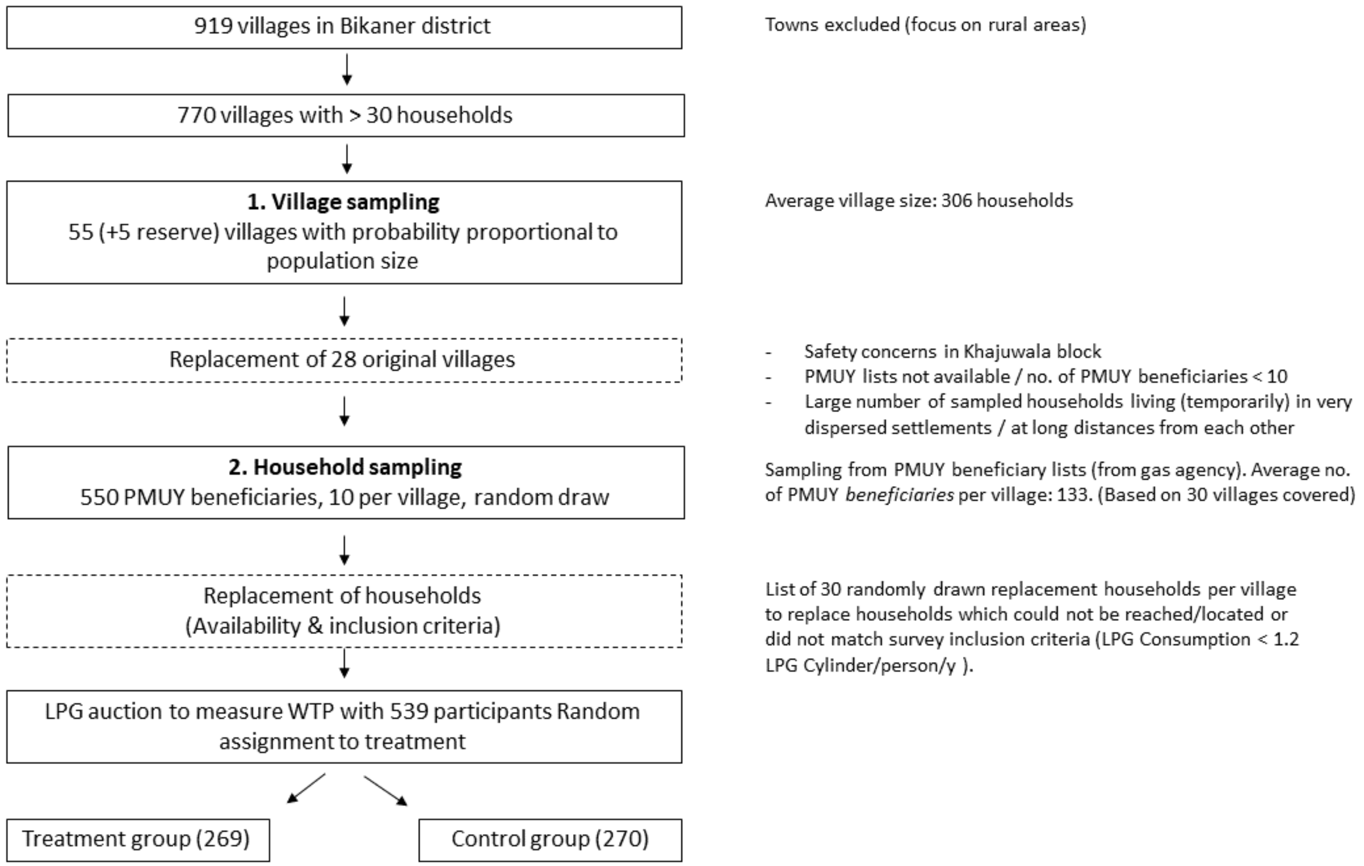

Supplement: S2 Appendix — (PDF) [file pone.0231931.s002.pdf]
