# Supplementary material for: Health awareness and the transition towards clean cooking fuels: Evidence from Rajasthan
Source: PLoS One. 2020 Apr 29;15(4):e0231931. doi: 10.1371/journal.pone.0231931 (PMC7190100; doi:10.1371/journal.pone.0231931)
Supplement: S3 Appendix — (PDF) [file pone.0231931.s003.pdf]

# Summary statistics and balance tests

**Table 1.** Means and tests of treatment-control covariate balance

| | Total mean | Control mean | Treatment mean | Difference b | t |
|---|---|---|---|---|---|
| Male | 0.08 | 0.09 | 0.07 | 0.01 | (0.62) |
| Age | 28.55 | 28.29 | 28.81 | -0.52 | (-0.73) |
| Education | 1.50 | 1.57 | 1.43 | 0.14 | (1.43) |
| Household size | 6.00 | 5.85 | 6.15 | -0.30 | (-1.53) |
| Hindu | 0.97 | 0.98 | 0.96 | 0.02 | (1.24) |
| Muslim | 0.03 | 0.02 | 0.04 | -0.01 | (-1.02) |
| BPL | 0.60 | 0.57 | 0.63 | -0.06 | (-1.27) |
| Expenditures | 6752.59 | 6740.38 | 6764.75 | -24.37 | (-0.07) |
| Land | 0.66 | 0.63 | 0.68 | -0.05 | (-1.15) |
| Asset index | -0.01 | -0.04 | 0.01 | -0.05 | (-0.38) |
| Refills | 0.91 | 0.90 | 0.92 | -0.02 | (-0.88) |
| LPG consumption | 0.24 | 0.23 | 0.25 | -0.01 | (-0.99) |
| Wood quantity | 45.75 | 47.63 | 43.85 | 3.78 | (1.17) |
| Dung quantity | 52.47 | 51.78 | 53.17 | -1.39 | (-0.38) |
| Random price | 339.94 | 336.41 | 343.48 | -7.06 | (-1.29) |
| Content | 0.45 | 0.46 | 0.44 | 0.01 | (0.53) |
| Voucher validity | 21.05 | 21.34 | 20.75 | 0.59 | (0.37) |
| Subsidy | 0.15 | 0.13 | 0.17 | -0.05 | (-0.96) |
| LPG convenience | 1.49 | 1.48 | 1.49 | -0.01 | (-0.20) |
| Distance | 0.46 | 0.48 | 0.44 | 0.04 | (0.91) |
| Refill cost | 0.90 | 0.91 | 0.90 | 0.01 | (0.31) |
| Fin. restriction | 0.77 | 0.79 | 0.75 | 0.05 | (1.26) |
| Food taste | 0.57 | 0.57 | 0.56 | 0.02 | (0.38) |
| Safety | 0.24 | 0.24 | 0.23 | 0.01 | (0.28) |
| $N$ | 539 | 270 | 269 | 539 | |

* $p < 0.1$, ** $p < 0.05$, *** $p < 0.01$

**Table 2.** Means and tests of treatment-control covariate balance for voucher owners

| | Total mean | Control mean | Treatment mean | Difference b | t |
|---|---|---|---|---|---|
| Male | 0.10 | 0.12 | 0.07 | 0.05 | (1.49) |
| Age | 28.31 | 27.97 | 28.65 | -0.67 | (-0.73) |
| Education | 1.52 | 1.55 | 1.50 | 0.05 | (0.37) |
| Household size | 5.89 | 5.75 | 6.03 | -0.29 | (-1.22) |
| Hindu | 0.97 | 0.99 | 0.94 | 0.05*** | (2.60) |
| Muslim | 0.03 | 0.01 | 0.05 | -0.05** | (-2.40) |
| BPL | 0.57 | 0.58 | 0.57 | 0.01 | (0.14) |
| Expenditures | 6511.90 | 6405.41 | 6619.86 | -214.46 | (-0.44) |
| Land | 0.66 | 0.67 | 0.65 | 0.01 | (0.24) |
| Asset index | -0.09 | -0.14 | -0.04 | -0.10 | (-0.65) |
| Refills | 0.91 | 0.89 | 0.93 | -0.04 | (-1.14) |
| LPG consumption | 0.25 | 0.24 | 0.26 | -0.02 | (-1.09) |
| Wood quantity | 46.15 | 47.54 | 44.72 | 2.82 | (0.68) |
| Dung quantity | 51.99 | 49.48 | 54.56 | -5.07 | (-1.08) |
| Random price | 305.43 | 301.51 | 309.40 | -7.89 | (-1.51) |
| Content | 0.43 | 0.42 | 0.44 | -0.02 | (-0.61) |
| Voucher validity | 20.61 | 20.42 | 20.82 | -0.40 | (-0.20) |
| Subsidy | 0.17 | 0.14 | 0.21 | -0.08 | (-1.14) |
| LPG convenience | 1.50 | 1.53 | 1.47 | 0.06 | (0.66) |
| Distance | 0.47 | 0.44 | 0.50 | -0.06 | (-1.08) |
| Refill cost | 0.88 | 0.87 | 0.90 | -0.03 | (-0.84) |
| Fin. restriction | 0.74 | 0.75 | 0.72 | 0.03 | (0.56) |
| Food taste | 0.53 | 0.56 | 0.51 | 0.06 | (0.97) |
| Safety | 0.25 | 0.26 | 0.24 | 0.02 | (0.43) |
| WTP for LPG | 389.83 | 382.88 | 396.91 | -14.03* | (-1.87) |
| Voucher value | 174.64 | 178.59 | 170.60 | 7.99 | (1.53) |
| *N* | 303 | 153 | 150 | 303 | |

* $p < 0.1$, ** $p < 0.05$, *** $p < 0.01$

**Table 3.** Variable definitions and summary statistics

| Variable | Definition | Count | Mean | Std. Dev. | Min | Max |
|---|---|---|---|---|---|---|
| **Dependent variables** | | | | | | |
| WTP | Willingness to pay for LPG | 539 | 356.79 | 71.34 | 200.0 | 750.0 |
| Voucher use | Dummy = 1 if individual used voucher before the household-specific expiry date | 296 | 0.35 | 0.48 | 0.0 | 1.0 |
| **Key explanatory variable** | | | | | | |
| Health information | Dummy = 1 if individual is exposed to health information | 539 | 0.50 | 0.50 | 0.0 | 1.0 |
| **Other variables** | | | | | | |
| Voucher | Dummy = 1 if individual received voucher | 539 | 0.56 | 0.50 | 0.0 | 1.0 |
| Voucher validity | Days until voucher expiry | 538 | 21.05 | 18.22 | 3.5 | 172.0 |
| Voucher value | Voucher value (INR) | 303 | 174.64 | 45.44 | 5.0 | 235.0 |
| Male | Dummy = 1 if individual is male | 539 | 0.08 | 0.27 | 0.0 | 1.0 |
| Age | Age of the individual | 539 | 28.55 | 8.18 | 18.0 | 65.0 |
| Education | Education(Categorical, levels 1-7) | 539 | 1.50 | 1.16 | 1.0 | 7.0 |
| Household size | Number of persons sharing one kitchen | 538 | 6.00 | 2.31 | 2.0 | 20.0 |
| Hindu | Dummy =1 if individual is Hindu | 539 | 0.97 | 0.17 | 0.0 | 1.0 |
| Muslim | Dummy =1 if individual is Muslim | 539 | 0.03 | 0.17 | 0.0 | 1.0 |
| BPL | Dummy = 1 if household holds a BPL card | 508 | 0.60 | 0.49 | 0.0 | 1.0 |
| Expenditures | Household consumption expenditures (INR/month) | 521 | 6752.59 | 4184.69 | 400.0 | 50000.0 |
| Land | Dummy = 1 if household owns land | 539 | 0.66 | 0.48 | 0.0 | 1.0 |
| Asset index | Weighted index of asset ownership | 539 | -0.01 | 1.42 | -1.4 | 6.6 |
| Refills | Dummy = 1 if household buys LPG refills | 539 | 0.91 | 0.29 | 0.0 | 1.0 |
| LPG consumption | Estimated LPG consumption HH (cylinder/month) | 489 | 0.24 | 0.15 | 0.0 | 1.7 |
| Wood quantity | Wood quantity used (kg/week) | 536 | 45.75 | 37.29 | 0.0 | 350.0 |
| Dung quantity | Dung quantity used (kg/week) | 537 | 52.47 | 42.11 | 0.0 | 350.0 |
| Content | Estimated content currently used cylinder (%) | 468 | 0.45 | 0.25 | 0.0 | 1.0 |
| Subsidy | Dummy = 1 if household buys | | | | | |

| | | | | | | |
|---|---|---|---|---|---|---|
| | subsidized cylinders | 232 | 0.15 | 0.35 | 0.0 | 1.0 |
| LPG convenience | Convenience LPG vs. trad. cooking | | | | | |
| | (1-Better, 2-Similar, 3-Worse) | 539 | 1.49 | 0.74 | 1.0 | 4.0 |
| Distance | Dummy = 1 if distance explains low LPG usage) | 539 | 0.46 | 0.50 | 0.0 | 1.0 |
| Refill cost | Dummy = 1 if refill costs explain low LPG) | 539 | 0.90 | 0.30 | 0.0 | 1.0 |
| Fin. restriction | Refill costs as main hindrance | | | | | |
| | to regular LPG consumption (respondents share) | 539 | 0.77 | 0.42 | 0.0 | 1.0 |
| Food taste | Dummy = 1 if taste of food explains low LPG) | 539 | 0.57 | 0.50 | 0.0 | 1.0 |
| Safety | Dummy = 1 if safety explains low LPG) | 539 | 0.24 | 0.42 | 0.0 | 1.0 |
| Severe effects | Dummy = 1 if aware of severe effects from IAP | 503 | 0.31 | 0.46 | 0.0 | 1.0 |
| Slight effects | Dummy = 1 if aware of slight effects from IAP | 503 | 0.53 | 0.50 | 0.0 | 1.0 |
| No effects | Dummy = 1 if not aware of any effects from IAP | 503 | 0.16 | 0.37 | 0.0 | 1.0 |
| IAP diseases | Share of six IAP-related diseases | | | | | |
| | correctly identified (in %) | 539 | 0.34 | 0.28 | 0.0 | 1.0 |
| All diseases | Share of ten diseases correctly | | | | | |
| | identified as either IAP-related or not | 539 | 0.51 | 0.15 | 0.1 | 0.9 |
| Observations | 539 | | | | | |

Sample restricted to respondents taking part in the WTP Experiment.

IAP = Indoor Air Pollution
