# Supplementary material for: Health awareness and the transition towards clean cooking fuels: Evidence from Rajasthan
Source: PLoS One. 2020 Apr 29;15(4):e0231931. doi: 10.1371/journal.pone.0231931 (PMC7190100; doi:10.1371/journal.pone.0231931)

Protocol and material for WTP elicitation and experiment

1 Protocols for WTP eliciting mechanisms and experiment

As explained in the main document, in order to ensure that survey participants would well understand the procedure leading to our measurement of WTP, we first ran the process with a relatively cheap item (a piece of soap) that respondents had to buy if their stated WTP was above a randomly drawn offer price. We then repeated the same procedure with a more expensive good (an LED bulb). This means that once the process was implemented for LPG, respondents had already gained significant experience with the procedure. In this Appendix we provide the protocol used for the WTP measurement of the piece of soap and the complete protocol for the LPG WTP experiment. The WTP measurement for the LED bulb was more or less identical to the one for soap, except for some reference to the experience the respondent already had with soap. It is therefore not shown here. The following questions and instructions were communicated verbally by the enumerator to the respondent. Occasional instructions in italics were directed to the enumerator. The enumerators carried these instructions on their mobile phone and the survey application of Qualtrics we had prepared took them automatically from one step to the next.

1.1 Protocol for measuring the willingness to pay for soap

1.1.1 Introduction

In this survey we will mainly ask you things about your energy use for cooking and lighting. In this context, we will also ask you to take a couple of decisions about real products. For this reason, before we start, we would like to go through a little exercise, so you can see how these decisions will work. I will now explain to you how it works.

In the following, you can purchase an item, as follows: First, you (participant) say the maximum amount you are ready to pay for it. The actual price will be unknown, but we will tell you the range of prices possible. We will then find out, at which price the good will be made available to you for purchase. To do so, we have prepared cards with prices within the possible range.

I will ask you to draw one card that will then show the relevant price. If this price is less than your originally stated maximum amount you would like to pay, then you will have to purchase the good at this price. If the price is higher than the amount you stated earlier, you cannot purchase it.

Maybe this procedure seems a bit complicated to you. But it makes sure that it is optimal for you to tell us your true willingness to pay. You cannot do better by stating a lower or higher price than you actually would be ready to pay.

Let us make an example for the procedure: You say you would be ready to pay up to 4 Rupees (INR), and the price on the card drawn is 7 INR The item will not be sold. If your price was 10 INR, the item is sold at 7 INR Note that if what you state is higher than the price drawn, this is an agreement to buy the good, and that if your stated amount is below the price, there will be no sale. Do you have any questions so far?

Let us now make the real run with a piece of soap. Are you willing to participate?

☐ Yes

☐ No

→ If *No* is selected, the survey ends at this point.

1.1.2 Bidding procedure

Here is a soap. The price at which it will be made available to you will be between 1 to 10 Rs, prices within this range are printed on the cards that you can see here. Now what is the maximum amount you are ready to pay to get it?

→ Participant states a price.

I would like to remind you that if you state an amount that is higher than the price which will be determined in a minute, this is an agreement to purchase the soap, and that if your amount is below the price there will be no sale. Would you like to adjust your amount now?
If YES, go back to last question and adjust amount
What is the maximum amount the participant is willing to pay? Please write down the price that was stated in INR

____

We now determine the price at which the good is made available for you in a random manner. Please turn one of the cards upside.

→ The participant turns up one of the number cards in front of her. The cards show the numerical values 2, 4, ...10.

What is the actual price on the card? Please select the actual price on the card in the drop-down list

---

→ The survey software calculates difference between bid (stated WTP) and offer price (randomly drawn price). Depending on the difference being positive or not, it redirects the enumerator to instruction A or B, with filled-in numerical values.

A THE RESPONDENT CANNOT BUY THE SOAP
Your **maximum amount** of [...] INR was lower than the actual price of [...] INR which you have drawn from the cards. This means the price you are ready to pay is not as high as the sales price we found here. Therefore you cannot buy the soap.

B THE RESPONDENT CAN BUY THE SOAP
You are ready to pay an amount of [...] INR This is higher than (or same) as the actual price of the soap, which you have drawn from the cards, which is [...] INR This means you will purchase the soap for the price of [...] INR now.

Did the participant agree to buy the soap?

☐ Yes

☐ No, because ________________

1.2 Experimental Protocol to measure the effect of health messaging on the WTP for LPG

1.2.1 Introduction

Now we would now like to carry out an exercise on LPG, which is similar to the ones with the soap and LED carried out before. We would like you to bid for one cylinder of LPG. You will have the chance to buy a cylinder at a price somewhat below 480 INR Let us remind you that this is about a real purchase and that your decision will be truly implemented.

Do you agree to participate? In case you wish to consult with someone else in the family, you can do so.

Wait in case the respondent wants to call another person to participate for the rest of the question

☐ Yes

☐ No

→ If *No* is selected, survey software redirects enumerator to section "Exit questions experiment".

1.2.2 Conditions

Please listen carefully to the conditions: The mechanism is basically the same as for the soap and the LED bulb. But as you can imagine, we cannot carry the gas cylinders with us. Therefore we will work with vouchers.

Voucher: The current subsidized price for a cylinder of LPG (subsidy comes on your bank account) is about 480 INR If the price we draw is below that (480), we give you a voucher which will cover the difference.

Time period: There is another important difference to a normal order: you have to use the remaining LPG more intensively than before, such that you collect the new cylinder earlier than usual or than you might have planned. For your case this concretely means, that the cylinder that we offer you must be collected before [...] days

---

→ We fixed a specific deadline for each household that would require this household to consume the remainder of the LPG in the cylinder currently in use twice as quickly than under normal circumstances. The deadline was determined by the survey tool based on the information about the family's existing LPG consumption and the remaining time for using up the current cylinder using the information provided at the outset in the screening questions. If this estimate could not be meaningfully interpreted (for instance, because the LPG connection was established only very recently), the household was directly asked to make a prediction on when they would need a refill and this prediction was halved to replace the estimate. The next working day after the end of this period constituted the deadline for the validity of the voucher.

1.2.3 Information intervention

Before we start, let me inform you that ...

→ Automated randomization through the survey app, directly displaying either the health frame or the alternative frame to the enumerator.

Present the information to the respondent and show the poster together with it.

→ The Enumerator presents the information (either of the frames) and shows the illustrating poster together with it. See all information material used for frames in section 2 of this Appendix.

1.2.4 Bidding procedure

Let us now turn to the bidding procedure. First let us find out what the maximum amount is that you are ready to pay for 1 cylinder of LPG, under the conditions I explained to you. Remember that it is optimal for you to tell us your true willingness to pay. You cannot do better by stating a lower or higher price than you actually would be ready to pay.

Now what is the maximum amount you are ready to pay for the cylinder?
Please note the price stated in INR

____

I would like to remind you that if you bid above the price we will just determine, this is an agreement to buy an LPG cylinder, and that if your bid is below the price you cannot buy LPG at special conditions. Would you like to adjust your bid now?
If yes go back to last question.

We now determine the price at which the good is made available for you in a random manner. Please turn one of the cards upside.

→ The participant turns up one of the number cards in front of her. The cards represent numerical values between 240 and 480, at intervals of 10 (starting from 245).

Note actual price from the card in INR

____

→ Survey software calculates difference between bid (stated WTP) and offer price (randomly drawn price). Depending on the difference being positive or not, it redirects the enumerator to instruction A or B, with filled-in numerical values.

A. LPG VOUCHER
You are ready to pay an amount of [...] Rs, this is higher than (or same) as the actual price, which you have drawn from the cards, which is [...] INR. This means you will be able to buy a cylinder of LPG at the price of [...] INR now.

We will make this possible by giving you a voucher, which covers the difference between this price and 480 [the normal subsidized cylinder price]. In your case this is 480 minus [offer price], equals [calculated voucher value] INR. For this we have an agreement with [name local distributor]. You can go there or order via phone/SMS and then use the voucher like cash. As we have explained before, there is a special condition of this voucher: You have to use the voucher to buy the next LPG cylinder before [calculated deadline] days.

Please take the voucher and fill it in:

- Name of interviewed person
- Her full address
- Voucher amount: (480 – [offer price] INR) , this is [calculated vouchervalue] INR
- Valid until: Today's date + [calculated time to used up current cylinder] days

Explain very clearly that they have to use the voucher until this date, because after this date it will not be valid anymore, the voucher will become useless.

Please enter the voucher number here:

____

Please take a photo of the voucher.

B. NO LPG Voucher
The amount you are ready to pay for 1 cylinder [...] INR is lower than the actual price of [...] INR which you have drawn from the cards. This means what you would like to pay is not enough to buy a new cylinder under these special conditions. Therefore, you cannot buy the LPG cylinder now.

1.2.5 Exit questions

Q1
Considering the impact on health, compared to traditional cooking stoves, the LPG-based cooking is:

☐ Better (1)

☐ Similar (2)

☐ Worse (please specify) (3): ________________

☐ Don't know (4)

→ Enumerator is directed to

Q2 if "Considering the impact on health, compared to traditional cooking stoves, the LPG-based cooking is" = Better (1)

Q3 if "Considering the impact on health, compared to traditional cooking stoves, the LPG-based cooking is" = Similar (2)

Q4 if "Considering the impact on health, compared to traditional cooking stoves, the LPG-based cooking is" = Don't know (4)

Q2
Let us talk a bit more about that. If cooking with firewood and/or dung affects the health, how severe is that? I will read out 2 statements now, please which one corresponds to your opinion:

☐ Cooking with dung cakes/firewood causes coughing and irritated eyes, but this health impact is not a severe problem.

☐ Cooking with firewood/dung cakes over a long time can cause very severe health problems.

☐ Other (please specify): ________________

Q3
Let us talk a bit more about that. I read out 2 statements now, please tell me which one corresponds to your opinion:

☐ No, I do not think that cooking with firewood and/or dung cakes causes any health problems.

☐ Cooking with dung cakes/firewood causes coughing and irritated eyes, but this health impact is not a severe problem.

Q4

Let us talk a bit more about that. I read out 3 statements now, please tell me which statement corresponds to your opinion:

- ☐ No, I do not think that cooking with firewood and/or dung cakes causes any health problems.
- ☐ Cooking with dung cakes/firewood causes coughing and irritated eyes, but this health impact is not a severe problem.
- ☐ Cooking with firewood/dung cakes over a long time can cause very severe health problems.

Q5

I am going to name lot of different health problems now. Some of them have to do with the smoke from the chulha, others don't have anything to do with it at all. For each one, could you tell me whether you think that cooking from firewood or dung cakes can make it more likely to suffer from this disease?

Disease	Yes	No
Arthrosis / Jodbandi	○	○
Heart diseases	○	○
Lung Cancer	○	○
Osteoporosis (bone atrophy)	○	○
Cataract / Motyaabind	○	○
Pneumonia	○	○
Problems for physical child development	○	○
Dengue fever	○	○
Diarrhea / Haija	○	○
Stroke / Aaghaat	○	○

Q6

Do you feel any discomfort from cooking with firewood and/or dungcakes? Please select the statement which fits best.

- ☐ No, I do not feel any discomfort.
- ☐ Cooking with dung cakes/firewood causes me to caugh more and have irritated eyes, but it is not a problem for me.
- ☐ I feel discomfort in my lungs and eyes and I have already experienced severe health problems, which have to do with the smoke from the chulha.
- ☐ Don't know.

2 Information material used for frames

While this appendix contains English versions of the visualizing posters and texts, the material used in the field was in Hindi language.

2.1 Health frame (text for enumerator and poster)

Before we start let me inform you that LPG is very different from firewood and dung cake regarding the health effects of these fuels. You have certainly observed that when cooking with the chulha – especially indoors and with bad ventilation – there is a lot of pollution in the air (*show picture of cooking woman*). According to studies from different universities and research institutions, this pollution causes many more health problems than may be directly observable for the person who cooks and her family. As opposed to what one may think, the effects are not limited to temporary coughing, tearing eyes and throat ache, but also include several severe diseases:

- Generally, many people in India die much earlier than normal from disease which is caused by air pollution from cooking with solid fuels.
- A large number of people, for instance, die prematurely due to a stroke. This occurs when blood flow to an area of the brain is cut off. Every 4th case of death from stroke is due to breathing in the polluted air over a long period of time.
- Similarly, indoor air pollution increases the risk of developing lung cancer or heart disease significantly. It is like smoking a very large amount of cigarettes every day, you can see on the picture what can happen to the lung (*show pictures lung diseases and heart diseases*).
- It also increases the chances of getting a cataract/motyaabind (*show picture eye diseases*). If untreated, cataract/motyaabind can lead to blindness.
- And it can hinder the development of (the) children. Women and small children are the most affected by the pollution. When small children die from acute lower respiratory infections like pneumonia, this is due to indoor air pollution in more than half of the cases (*show picture development of child*). Of course, ventilation helps to reduce these risks. Having an open window and a chimney hood or cooking outside is therefore helpful. But according to available academic studies, the remaining risks are often considerable and should not be underestimated. When cooking on a chulha, the danger of being hit by the severe diseases mentioned above is usually still much higher than otherwise.

Fig 1. Visualizing poster health frame.

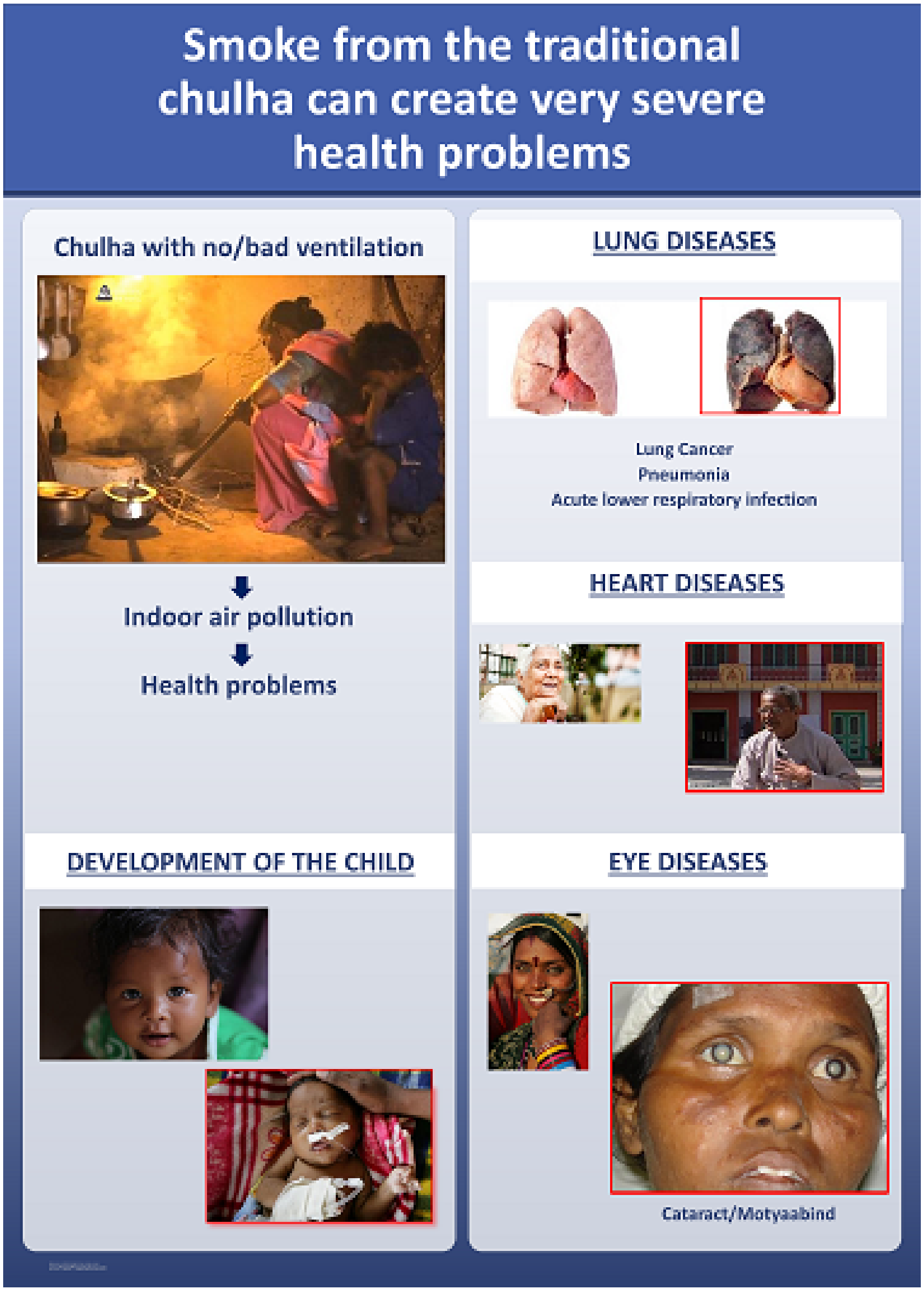

2.2 Alternative frame (text for enumerator and poster)

Before we start, let me give you some information about how liquefied petroleum gas or LPG, your cooking gas, is produced. LPG is a fossil fuel. Sometimes it is recovered naturally, directly from the ground. Another way of producing LPG is by refining it from crude oil. Crude oil is a thick and black liquid. It is a mixture of different chemicals which can be used as fuel because they burn well. Most crude oil is found by drilling down through rocks on land or off-shore at the bottom of the ocean.

- Look, we have a picture of an oil field off the coast of Mumbai. The oil gets pumped up from a deep hole in the ocean floor (*show picture of oil field off the coast of Mumbai*).
- Crude oil cannot be used as a fuel as it is. Therefore, the crude oil must be transported to a so-called oil refinery as a first step. This can best be done through a crude oil pipeline, which pumps the crude oil from the oil field to a refinery (*show picture of crude oil pipe*). This pipeline transports crude oil from the Barmer district, Rajasthan to Salaya, Gujarat.
- At the oil refinery, the crude oil is heated and then distilled to separate it into different petroleum products (*show picture of oil refinery*). These include gasoline for cars, ship fuel and the petroleum gas used for cooking.
- But gas takes up a lot of space. To make storage easier, the gas is liquefied by compressing with high pressure. This is why your cooking gas is called liquefied petroleum gas or LPG.
- Then the liquefied gas is transported to a bottling plant. There it gets filled into the cylinders that you know (*show picture of bottling plan*). They are small enough for relatively easy transport. Since the gas is still liquid, it does not take up too much room.
- As a last step, LPG distributors deliver the LPG cylinders to customers in local markets (*show picture of delivery*).
- In some major cities, households do not have to buy the LPG bottled up in cylinders, but instead receive gas through a pipeline in their kitchen (*show picture of woman with stove and gas pipeline*).
- If you release the liquid from the cylinder by turning on your appliance, it turns back into gas.

Fig 2. Visualizing poster alternative frame.

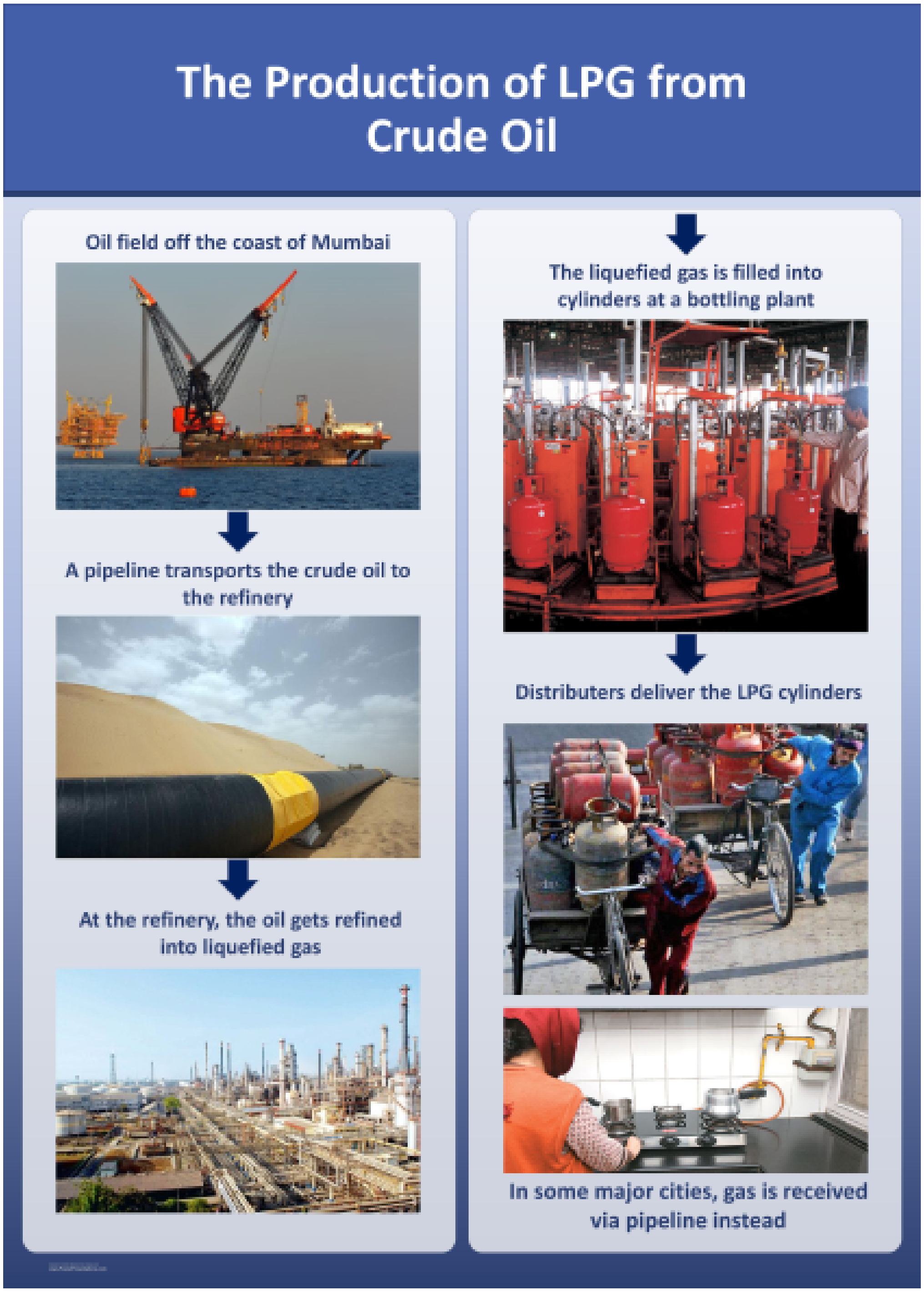

Supplement: S4 Appendix — (PDF) [file pone.0231931.s004.pdf]
