# Supplementary material for: Health awareness and the transition towards clean cooking fuels: Evidence from Rajasthan
Source: PLoS One. 2020 Apr 29;15(4):e0231931. doi: 10.1371/journal.pone.0231931 (PMC7190100; doi:10.1371/journal.pone.0231931)
Supplement: S5 Appendix — (PDF) [file pone.0231931.s005.pdf]

Offer price distribution and voucher use

Offer Prices in the WTP eliciting mechanism

Enumerators determine the offer prices p_d used in the WTP eliciting mechanism by drawing a random piece from a set of price cards covering the range from 245 to 475 INR in steps of 5 INR.

The choice of the price range is based on the following reflection: Even if a household's currently used cylinder was full at the time of the survey and even if its demand for LPG was completely inelastic (i.e., it does not speed up consumption due to price reductions), it would be left with a maximum of 50% of the cylinder content (market value = 240 INR) at the expiry date of the voucher, i.e., when the cylinder is to be replaced by a full one. All households should thus accept to replace their currently used cylinder by a full one if they are given a compensation $C = 480 - 240 = 240$ INR, and a higher discount should not be necessary in our context.

Fig 1 shows the empirical distribution of the prices. While prices were drawn from the full range of possible values, their distribution is right-skewed. Offer prices below the mean (339) are more frequent than prices that are higher than this average. This is surprising as an approximately uniform distribution of offer prices should have been expected. A chi-square test comparing the observed frequencies to the expected frequencies under a discrete uniform distribution clearly rejects the null-hypothesis that these distributions are equal (p=0.000).

Fig 1. Distribution of offer prices.

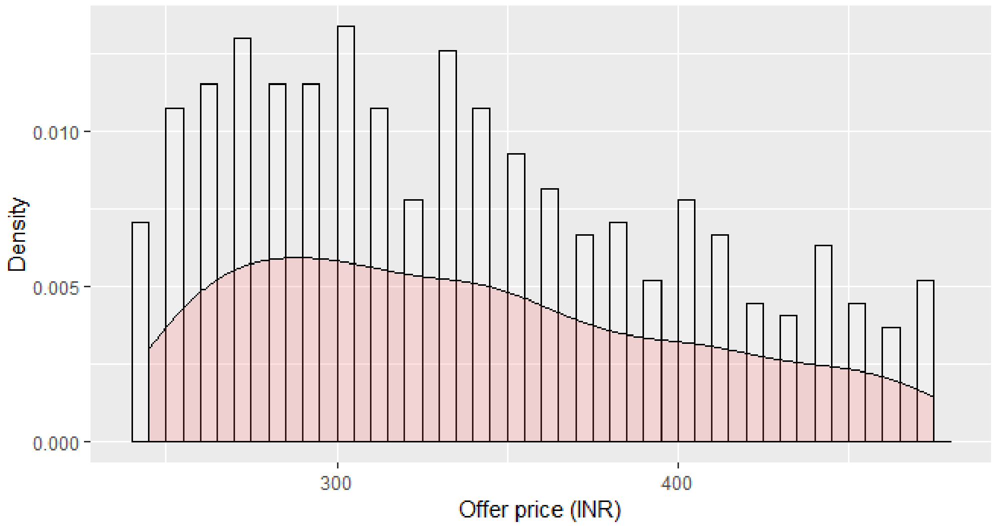

Histogram with heights of the bars representing observed frequencies of offer prices and density curve as approximation of the proportion of values in certain price ranges.

This raises some doubts regarding the random selection of offer-prices. It cannot be excluded for instance that, in some cases, enumerators made the selection only among higher discount values in order to provide extra benefits to the household. However, since WTP is measured before offer prices are drawn, this should not affect our main results.

Offer Prices and voucher use probability

This section examines the effect of the randomly determined offer price in more detail. Table 1 shows the results of a logistic regression estimation that includes the voucher value, i.e., the offered price discount D $(= p_m - p_d)$ as a continuous variable (odds ratios displayed).

Table 1. Joint effect of health information on voucher use.

	(1)	(2)	(3)	(4)
Health message	1.444*	1.724**	7.633**	11.421**
	(0.095)	(0.020)	(0.031)	(0.029)
Discount (per 20 INR)		1.360***	1.516***	1.564***
		(0.000)	(0.000)	(0.000)
Discount X Health message			0.843*	0.822
			(0.097)	(0.106)
Male				2.906**
				(0.023)
Content				0.548
				(0.407)
Voucher validity				0.996
				(0.680)
Asset index				1.122
				(0.219)
Land				1.221
				(0.481)
LPG distance				1.024*
				(0.050)
Fin. restriction				1.400
				(0.314)
Education				1.019
				(0.874)
Age				0.981
				(0.289)
Household size				0.889*
				(0.086)
Months since LPG adoption				1.024
				(0.181)
Constant	0.203***	0.017***	0.006***	0.007***
	(0.000)	(0.000)	(0.000)	(0.000)
N	532	531	531	449
Area under the ROC curve	55%	73%	73%	77%

Logit models with odds ratios,* $p < 0.1$, ** $p < 0.05$, *** $p < 0.01$. p-values in parentheses. Lack of data on the additional variables included in Col. 2-4 leads to a reduction in the number of observations.

Col. 1 reports the odds ratio for the uncontrolled comparison between treatment and control group as a baseline (corresponding to Table 5, Col. 1 in the main part of the text). As discussed in the section "Joint effect of health messaging on voucher use", the estimate indicates that the odds of using the voucher are 1.44 times larger for a household that received the health messaging than for a household that did not. Col. 2 shows that the treatment effect is not much affected by the additional inclusion of the offer price. The odds ratio increases from 1.44 to 1.72. This corresponds to a change in the predicted probability of voucher use from 6 percentage points for the model in Col. 1 to 8 percentage points for the model in Col. 2. Furthermore, the estimate becomes more precise. Generally, the inclusion of prices substantially increases the precision of the model and its capacity to correctly predict the use or non-use of the voucher. The effect on the quality of the prediction is similar to the effect of the inclusion of price dummies in Table 5 of the main text.

The discount itself has a robust and also quite sizeable effect. On average, a price reduction of 20 INR increases the odds of using the voucher by a factor of 1.36. This corresponds to an increase in the predicted probability of using the voucher by 4 percentage points.

In Col. 3 and 4 we allow the price reduction to interact with the treatment effect and add further controls. Measured in terms of odds ratios, the interaction term is just at the border of significance. However, this is not very meaningful because, when the discount is close to zero, almost no respondent uses the voucher so that even a tiny absolute effect of health messaging appears like a huge effect in relative terms.

Rather than to interpret the interaction term in Table 1, we thus move to a graphical illustration of the probability of voucher use for different treatment conditions and different discount values. Using the regression in Col. 4, Fig 2 shows how the predicted probability of voucher use increases with rising discounts (depicted in steps of 20 INR) for both the treated and the untreated. Comparing the lines for these two groups, we find no systematic reduction in the distance between treated and non-treated for increasing voucher values. The marginal effect of the discount is also not changed by the treatment (p=66%, see Stata code in Appendix S6). On average, across all observed values in the sample, the effect of health messaging is about 10 percentage points. Differences are most clearly significant for intermediate voucher values.

Fig 2. Predicted Probability of voucher use.

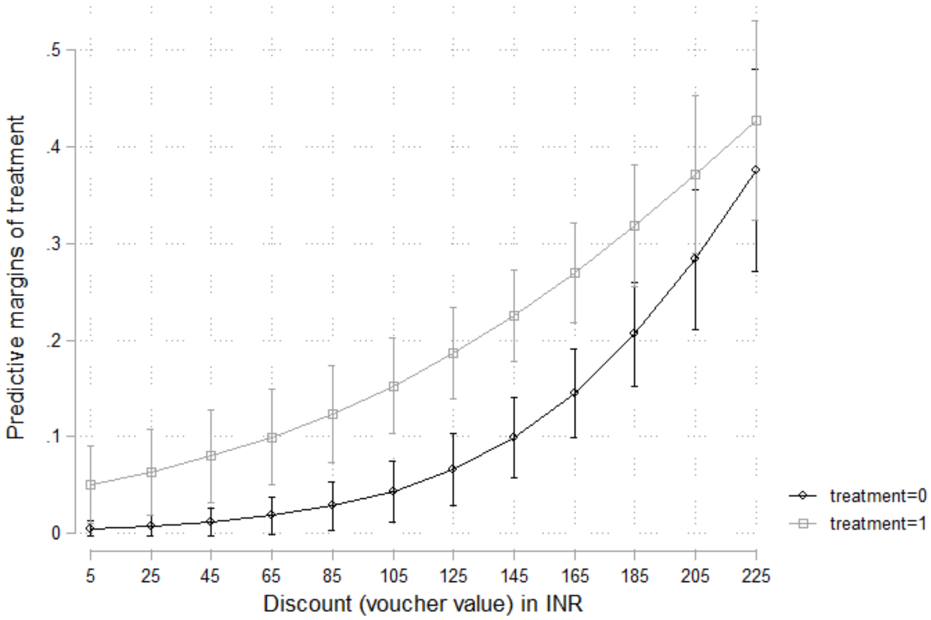

Logistic regression model as estimated in Col. 4 of Table 1 and 90% CIs

Comparing the price effect and the treatment effect

Fig 2 also allows us to compare the change in the predicted probability of voucher use driven by the treatment to the change induced by different discount values. For very low voucher values (at the left of the graph) health messaging increases the probability of voucher use by about 5 percentage points. To reach the same effect size, the voucher value must be increased by 100 INR (from 5 to 105 INR). In the middle part of the graph, the treatment effect appears somewhat stronger, but the slope of the curve of the non-treated is steeper, implying that a further discount matters more, too. At a discount value of 125, for instance, the effect of the health treatment is almost 15 percentage points, but the same effect can be reached by increasing the discount by 60 INR, i.e., a lesser amount than before, from 125 to 185 INR. On average across the range of observed values in the sample, increasing the discount by 40 INR (=8.3% of the current subsidized price of a new cylinder) increases voucher use by about 10 percentage points, and thus corresponds to the effect size of health messaging within the same model (Table 1, Col. 4).
