# Supplementary material for: Health awareness and the transition towards clean cooking fuels: Evidence from Rajasthan
Source: PLoS One. 2020 Apr 29;15(4):e0231931. doi: 10.1371/journal.pone.0231931 (PMC7190100; doi:10.1371/journal.pone.0231931)
Supplement: Attachment — Submitted filename: Response to the reviewers.pdf [file pone.0231931.s009.pdf]

PONE-D-19-28805R1
Health awareness and the transition towards clean cooking fuels: Evidence from Rajasthan
PLOS ONE

Response to the reviewer

We are happy to hear that our revisions have fully addressed all comments, except some further changes you would like regarding the statistical modelling and the corresponding presentation of our results.

The issues raised were the following:

First, theoretically the use of a probit/logit model is the correct specification for a probability study. In fact, the use of a logit model makes interpretation much easier by simple exponentiation of beta coefficients in the logistic specification. Empirically, the authors themselves report that running a probit model (it will be the same result for logit), they obtain similar results that "are even more precise". Why are the authors choosing to present the less precise (poorer-goodness-of-fit) results in the main body of the paper and have the more precise ones buried in a Supplementary file? Beyond correctness in modeling and accuracy in empirical estimation, running a logistic regression is simple to do in any standard statistical software package. The interpretation of exponentiated beta logit coefficients is very straightforward. The authors should remove the current results from the' linear probability' model and replace them accordingly with those from a logistic regression.

We have now revised the econometric modelling and the presentation in the text accordingly.

Throughout the paper, we now use logit models to present the results for estimations with binary outcome variables. We keep the code for linear probability models for comparison in Appendix S6, in case the readers are interested in checking the robustness of our results to the modelling approach.

The specific adjustments are as follows:

- Section “Effect of health messaging on voucher use among voucher owners”:
  We present the results in terms of odds ratios in Table 3 and in terms of predicted probabilities in Table 4. The corresponding discussion of results (lines 623-698) is adjusted accordingly.

- Section “Joint effect of health messaging on voucher use”:
  We present the results in terms of odds ratios in Table 5 and in terms of predicted probabilities in Table 6. The corresponding discussion of results (lines 700-739) is adjusted accordingly.

- Section “Testing the information channel”:
  In Table 7. “Treatment effect on health-awareness”, Col. 1 we now show average marginal effects based on a logit model. We do not show odds ratios here because the two other models in this table also show marginal effects as they relate to linear models for non-binary dependent variables. This facilitates the comparison across columns.

The discussion remains essentially unchanged because the average marginal effects from the logit model are almost completely identical with the coefficient estimates of the linear probability model previously shown in Col. 1 of this table.

- Appendix S5, Section “Offer Prices and voucher use probability:” For the regression including an explicit modelling of the role of offer prices explicitly, we now use a logistic model, too. Thus, we present our results in Table 1 in terms of odds ratios, and the graphical illustration of changes in the predicted probabilities in Figure 2 is based on the logit model, too. The corresponding discussion of results has been adjusted and expanded to explain the rather tricky interpretation of the interaction between the discount and the health messaging. As we explain there, the graphical presentation as well as the presentation in terms of changes in predicted probabilities appear most instructive in this context. This discussion appears important to us given its relevance for policy making. (Only the results of this discussion are mentioned in the main document, see lines 737-740).

- Abstract and conclusion: Due to marginal changes in our results small text adjustments were required regarding the comparison of a price reducing policy (discount) and health messaging (see 2nd last sentence in the Abstract, and Conclusion, lines 837-838).

Please note that the changes mentioned above (i.e., the changes in response to the review decision of 18 February 2020) are highlighted in yellow color in the Word document in order to distinguish them from prior changes in response to the first round of reviewer comments. All changes to the originally submitted version (from December 2019) are still visible in the track-changes mode.
