# Supplementary material for: Health awareness and the transition towards clean cooking fuels: Evidence from Rajasthan
Source: PLoS One. 2020 Apr 29;15(4):e0231931. doi: 10.1371/journal.pone.0231931 (PMC7190100; doi:10.1371/journal.pone.0231931)
Supplement: Attachment — Submitted filename: Health_awareness.docx [file pone.0231931.s007.docx]

**Health awareness and the transition towards clean cooking fuels: Evidence from Rajasthan**

In my opinion, this is an interesting paper that adds new evidence to the domain literature. However, I would like to see the following major and minor revisions to recommend it for publication.

General: Different sections are not labelled. Do that!

Major

- The experiment does not support the conclusion regarding heterogeneous treatment effects for male and female respondents. Male respondents were not basically your experiment units. You include males only because the selected women are unavailable or can not answer for cultural or some other reasons.
- I strongly recommend the literature section (lines 45-163) to be reorganized. It appears very fragmented with several unlabelled sections.
- It is important to emphasize that the household does not derive utility from LPG, but from the energy services that use LPG as an input. You need to modify your theoretical model and show that LPG demand is a derived demand.

Minor

- Abstract …..about 2.9 billion people ………….. Provide a time reference? When is this? And source?
- Abstract and also in the main text, ….health messaging/health information consider to write this consistently. I personally prefer to use either of them throughout the paper.
- Line 5, is this due to only household air pollution? Make it clear.
- Line 15, …Fuel stacking ….. mention that it means multiple fuel use.
- Line 25, …. Indeed, our interviews demonstrate…… is this interview prior to your study? with the study subjects? And did you include health information based on this interview result? I expect you to first introduce what your study is about.
- Line 31, …some general information ….. Did you also give this general information to the treatment group? Make it clear. This is where your reader should know the difference between the treatment and control groups.
- Lines 32-35, ….. Make your hypothesis very clear. What have you hypothesised regarding the effect of the treatment on the outcome variables? It is not stated plainly, especially for the first variable.
- Number your sections, see general comment. As it appears now, it is difficult to make cross-referencing.
- Lines 52-55, …..majority of households underestimate the severity of the risks but consider information demonstrating severity salient .......Isn't this counter intuitive given you already said that "majority of households recognize that there are some health hazards related to indoor air pollution"? consider revising.
- Line 61…Mixed findings ….. Your review does not show that the findings in the literature are mixed. I cannot see a study that find that health information increases WTP for clean cooking solutions or adoption of improved stoves.
- Lines 64-68, repeated sentence
- Line 78, …..Tibet….where is it? China?
- Line 86, ….does not provide consistent evidence…. What do you mean by this? information did not matter?
- Line 89, mention these studies (RCTs)
- Line 134, did not you already mention on line 131 that load can arranged to buy the first cylinder? / and here free of charge/?
- Line 146, I think, this is what you have done and will contribute to the literature. I suggest that you should elaborate this in the introduction.
- Line 167, x includes …….revise/rewrite this sentence
- Lines 246-247…….It would be nice it you include a map of your study area.
- Line 260 …..electricity is more widespread …….consider revising/rephrasing it.
- Lines 263-267, sex ratio is over discussed. If you feel it helps to read your paper, write it in a footnote. The discussion diverts attention to a different topic.
- Line 282, your respondents are not in the households´ decision-making position, and therefore, your discussion should be streamlined taking this into account. I can see that you try to caution the reader, but you still appear to make strong conclusions. I strongly advise you to tone down your statements related to female-male treatment effects. See major comment 1.
- Line 347, the way you illustrate the diseases (presentation of pictures) per se may affect respondents’ mindsets and hence decisions. I think you should elaborate that the pictures are not disturbing or very sensitive to have such effects.
- Line 548, First indicate what each column in the table shows and then proceed to the explanations to make your table more readable.
- Table 2, column 3 and 4….it is not clear why the number of observations has decreased.
- Line 581, …land ….. is this land ownership regardless of size?
- Line 615 …info…. write information in full
- Line 706-707….I am sceptical regarding your calculation here. Intuitively, the demand for LPG (probability of voucher use) should not linearly increase from 4 to 10% when discount increases from 20 to 50 INR. This is because households will not keep linearly substituting other consumptions to LPG as they will experience satiation from increasing consumption of LPG (Marginal utility of LPG consumption would fall.). At least, that is what your theoretical model implies. You cannot establish the equivalence, and if you do, you should make note of this.
- Line 744-748….repeated sentence
- Line 788…. Why is it particularly high in India?
- Line 828…..important for what?
