# Supplementary material for: Health awareness and the transition towards clean cooking fuels: Evidence from Rajasthan
Source: PLoS One. 2020 Apr 29;15(4):e0231931. doi: 10.1371/journal.pone.0231931 (PMC7190100; doi:10.1371/journal.pone.0231931)
Supplement: Attachment — Submitted filename: Response to the reviewers.docx [file pone.0231931.s008.docx]

PONE-D-19-28805
Health awareness and the transition towards clean cooking fuels: Evidence from Rajasthan
PLOS ONE

**Response to the editor and the reviewers**

Dear Prof. Michaelowa,

Thank you for submitting your manuscript to PLOS ONE. After careful consideration, we feel that it has merit but does not fully meet PLOS ONE’s publication criteria as it currently stands. Therefore, we invite you to submit a revised version of the manuscript that addresses the points raised during the review process.

The authors should address all issues raised by two anonymous reviewers. In particular, any conclusions about gender-effects should be closely considered given some of the inherent limitations of their experiment.

*Thank you very much for your advice. We have now revised the document in response to the reviewer comments, with a particular focus on the interpretation of gender-effects. For details, please see our response to the individual reviewers.*

Although it was mentioned by one reviewer, there is no need to number subsections. However, the authors should consider a mode descriptive heading for 'Appendix 1'.

*We now use the heading “Mathematical derivations for the theoretical model”.*

Given the nature of this research the authors should be fully compliant by making all experimental (laboratory) protocols available.

*We now present the full protocol of the WTP elicitation mechanisms and of the experiment as supporting information. It complements the information material used for the intervention in the file S4 Appendix 4. Consequently, we changed the heading of Appendix 4 to “Protocol of WTP elicitation mechanisms and experiment.”*

*The manuscript refers to this protocol in the sections on the experimental set-up and WTP elicitation. As a consequence, certain details in the section on the empirical procedure were not required in the main manuscript anymore, and could be removed to make the manuscript more concise.*

We would appreciate receiving your revised manuscript by Feb 17 2020 11:59PM. When you are ready to submit your revision, log on to https://www.editorialmanager.com/pone/ and select the 'Submissions Needing Revision' folder to locate your manuscript file.

If you would like to make changes to your financial disclosure, please include your updated statement in your cover letter.

To enhance the reproducibility of your results, we recommend that if applicable you deposit your laboratory protocols in protocols.io, where a protocol can be assigned its own identifier (DOI) such that it can be cited independently in the future. For instructions see: http://journals.plos.org/plosone/s/submission-guidelines#loc-laboratory-protocols

Please include the following items when submitting your revised manuscript:

- A rebuttal letter that responds to each point raised by the academic editor and reviewer(s). This letter should be uploaded as separate file and labeled 'Response to Reviewers'.
- A marked-up copy of your manuscript that highlights changes made to the original version. This file should be uploaded as separate file and labeled 'Revised Manuscript with Track Changes'.
- An unmarked version of your revised paper without tracked changes. This file should be uploaded as separate file and labeled 'Manuscript'.

Please note while forming your response, if your article is accepted, you may have the opportunity to make the peer review history publicly available. The record will include editor decision letters (with reviews) and your responses to reviewer comments. If eligible, we will contact you to opt in or out.

*Thank you very much for these details; the different documents have been prepared as requested.*

We look forward to receiving your revised manuscript.

Kind regards,

Francisco X Aguilar
Academic Editor
PLOS ONE

Journal Requirements:

1. When submitting your revision, we need you to address these additional requirements.

Please ensure that your manuscript meets PLOS ONE's style requirements, including those for file naming. The PLOS ONE style templates can be found at

http://www.journals.plos.org/plosone/s/file?id=wjVg/PLOSOne_formatting_sample_main_body.pdf and http://www.journals.plos.org/plosone/s/file?id=ba62/PLOSOne_formatting_sample_title_authors_affiliations.pdf

2. We note that you have stated that you will provide repository information for your data at acceptance. Should your manuscript be accepted for publication, we will hold it until you provide the relevant accession numbers or DOIs necessary to access your data. If you wish to make changes to your Data Availability statement, please describe these changes in your cover letter and we will update your Data Availability statement to reflect the information you provide.

*We now provide the dataset and do-file required to replicate all study findings reported in this article as Supporting Information S6 Dataset. A cross-reference to the material in the manuscript is included in the subsection “Effect of health messaging on voucher use among voucher owners”.*

3.  Thank you for stating the following in the Acknowledgments Section of your manuscript:

[We are grateful for the support of the Indian Ministry of Petroleum and Natural Gas, 839
Indian Oil Corporation Ltd. and all concerned field managers and LPG gas distributors 840
in Bikaner district in the implementation of this research. We also acknowledge the 841
contribution to the data collection by Prof. Rajesh Sharma and a team of students at 842
the SKRAU Agricultural University Bikaner. We gratefully acknowledge a Scholar 843
Exchange Grant awarded by the Indo-Swiss Joint Research Programme in the Social 844
Sciences jointly funded by the Indian Council for Social Science Research and the Swiss 845
State Secretariat for Education, Research and Innovation. Finally, we thank Lorenzo 846
Casaburi, Paula Castro, Ipsita Das, Jan-Walter De Neve, Sebastian Fehrler, Robert 847
Huber, Michael Grimm, Lennart Kaplan, Stefan Klonner, Olexiy Kyrychenko, Jorg 848
Peters, Massimo Phillipini, Atonu Rabbani, Alfonso Sanchez, Kirk R. Smith and 849
Johannes Urpelainen for their insightful comments that have helped us to improve the 850
draft of this paper.].

i) We note that you have provided funding information that is not currently declared in your Funding Statement. However, funding information should not appear in the Acknowledgments section or other areas of your manuscript. We will only publish funding information present in the Funding Statement section of the online submission form.

*We removed any funding related information from the acknowledgements section (see revised acknowledgement section in the manuscript). The only external funding we received came through the mentioned scholar exchange grant, which is financed by public research funding programs in both India and Switzerland. Apart from the grant, only resources from our own university budget have been used.*

*There is (and was) no undeclared funding information. This must have been a misunderstanding, possibly because we used the word “support” in the acknowledgements. To implement our study, we needed an agreement from the Indian Oil ministry and Oil marketing companies, and we had asked them to help us in making sure that the local LPG distributors would accept our vouchers and provide us the relevant information on the government’s program coverage that allowed us to draw our household sample. Based on the ministry’s support, we were then indeed able to invite the relevant distributors to a workshop, where we explained the research project and asked them for inputs on the practical details of the implementation on the ground. There was no financial contribution from these partners. We adjusted the acknowledgement section to clarify this point, i.e. we now state that we are grateful for the “facilitation” of the research implementation (instead of “support” in the implementation of the research).*

ii) Please remove any funding-related text from the manuscript and let us know how you would like to update your Funding Statement. Currently, your Funding Statement reads as follows:

 [The project benefitted from a scholar exchange grant provided to K.M. and P.D. by the Indo-Swiss Joint Research Programme in the Social Sciences (Call 2015) jointly funded by the Indian Council for Social Science Research and the Swiss State Secretariat for Education, Research and Innovation (https://www.unil.ch/international/home/menuguid/evenements/archives/indo-swiss-programme.html).
The funders had no role in study design, data collection and analysis, decision to publish, or preparation of the manuscript.].

iii) Additionally, because some of your funding information pertains to commercial funding, we ask you to provide an updated Competing Interests statement, declaring all sources of commercial funding.

*As clarified above, there was no commercial funding. We therefore wish to leave the Funding Statement unchanged.*

iv) In your Competing Interests statement, please confirm that your commercial funding does not alter your adherence to PLOS ONE Editorial policies and criteria by including the following statement: "This does not alter our adherence to PLOS ONE policies on sharing data and materials.” as detailed online in our guide for authors  <http://journals.plos.org/plosone/s/competing-interests>.  If this statement is not true and your adherence to PLOS policies on sharing data and materials is altered, please explain how.

*Please see comment above.*

v) Please include the updated Competing Interests Statement and Funding Statement in your cover letter. We will change the online submission form on your behalf.

*Please see comment above.*

Additional Editor Comments (if provided):

Two expert reviewers have identified various shortcomings in the present version of the manuscript. These should be addressed fully in a revised version for potential publication.


[Note: HTML markup is below. Please do not edit.]

Reviewers' comments:

Reviewer's Responses to Questions

**Comments to the Author**

1. Is the manuscript technically sound, and do the data support the conclusions?

The manuscript must describe a technically sound piece of scientific research with data that supports the conclusions. Experiments must have been conducted rigorously, with appropriate controls, replication, and sample sizes. The conclusions must be drawn appropriately based on the data presented.

Reviewer #1: Partly

Reviewer #2: Partly

2. Has the statistical analysis been performed appropriately and rigorously?

Reviewer #1: Yes

Reviewer #2: Yes

3. Have the authors made all data underlying the findings in their manuscript fully available?

The PLOS Data policy requires authors to make all data underlying the findings described in their manuscript fully available without restriction, with rare exception (please refer to the Data Availability Statement in the manuscript PDF file). The data should be provided as part of the manuscript or its supporting information, or deposited to a public repository. For example, in addition to summary statistics, the data points behind means, medians and variance measures should be available. If there are restrictions on publicly sharing data—e.g. participant privacy or use of data from a third party—those must be specified.

Reviewer #1: Yes

Reviewer #2: Yes

4. Is the manuscript presented in an intelligible fashion and written in standard English?

PLOS ONE does not copyedit accepted manuscripts, so the language in submitted articles must be clear, correct, and unambiguous. Any typographical or grammatical errors should be corrected at revision, so please note any specific errors here.

Reviewer #1: Yes

Reviewer #2: Yes

5. Review Comments to the Author

Please use the space provided to explain your answers to the questions above. You may also include additional comments for the author, including concerns about dual publication, research ethics, or publication ethics. (Please upload your review as an attachment if it exceeds 20,000 characters)

Reviewer #1:

In my opinion, this is an interesting paper that adds new evidence to the domain literature. However, I would like to see the following major and minor revisions to recommend it for publication.

*Let us first thank you for your thoughtful comments and advice, which have helped us in revising the manuscript. Our response to the specific revisions that you suggest are detailed below.*

General: Different sections are not labelled. Do that!

*We have ensured that the manuscript follows the PLOS ONE style, as per the editor’s explicit advice on this point.*

Major

- The experiment does not support the conclusion regarding heterogeneous treatment effects for male and female respondents. Male respondents were not basically your experiment units. You include males only because the selected women are unavailable or can not answer for cultural or some other reasons.

*Thank you for the important comment with which we are in full agreement. We definitely do not wish to stretch our interpretation beyond what the study design allows us to do. Our intent is to flag some of the preliminary questions on gender relations, which seemed to be emanating from the empirical analysis. These may be of interest for subsequent research. We have accordingly attempted to carefully revise the document to reflect this point. The major instances of revision are as follows:*

*- In the abstract, the sentences related to heterogeneous gender effects have been replaced by the following statement: “Finally, our study raises some interesting questions about gender-related effects that would be worth consideration in future research.”*

*- We avoid the term “heterogeneous treatment effects” regarding gender throughout the paper.*

*- We systematically caution the reader that statements made in this context hold only for the specific group of men included in our sample.*

*- In the discussion, we clarify consistently* *that our findings are exploratory and suggestive.*

*- In the conclusions, we replaced the extensive discussion of gender effects by the following brief statement: “Our empirical estimation was not designed to estimate heterogeneous treatment effects between women and men. Nevertheless, our study suggests some potentially relevant and partially unexpected gender differences that call for further investigation in future work. Independently of these results, given that women often lack decision-making power on major purchases, knowledge building should not target women alone.”*

- I strongly recommend the literature section (lines 45-163) to be reorganized. It appears very fragmented with several unlabelled sections.

*It is true that we have had some difficulties with the presentation of the different strands of the literature since none of them addresses our topic directly, but many are relevant in a broader sense. Following your advice, we have now rewritten large parts of the text in this section. While we have not changed the order of subsections, we have attempted to clarify the flow of the argument. To do so, we inserted a brief overview of the section and reorganized major parts of the discussion within different subsections. We believe that this makes the literature review both clearer and easier to read. Thank you!*

- It is important to emphasize that the household does not derive utility from LPG, but from the energy services that use LPG as an input. You need to modify your theoretical model and show that LPG demand is a derived demand.

*Thank you for highlighting this issue. Right at the beginning of the theoretical section, we now clarify this point: consumers derive utility from energy services that can be provided by different fuels, and these fuels are imperfect substitutes as they also differ on a number of other utility-relevant factors, notably health.*

*The current formulation of the formal model leads directly to the derivation of the hypotheses to be examined, thereby validating its appropriateness as a theoretical depiction of the context we seek to empirically examine. The focus is on the choice between LPG and other goods (notably other fuels) from the individual household’s point of view, rather than an estimation of overall energy demand for the household. In the formal model, LPG therefore enters the household’s utility function directly.*

Minor

- Abstract …..about 2.9 billion people ………….. Provide a time reference? When is this? And source?

*We clarified the year and rounded the number to 3 billion. We hope that this makes the sentence general enough not to require a citation, which we would like to avoid in the abstract. For those interested in the source, we now pick up the sentence with the more exact number again as the first sentence of the main text along with the relevant reference.*

- Abstract and also in the main text, ….health messaging/health information consider to write this consistently. I personally prefer to use either of them throughout the paper.

*We now consistently use the term “health messaging” for the treatment. The term “health information” still appears in the paper in some places, but only where it does not directly refer to the treatment (i.e., contexts such as “health information is more salient for…”).*

- Line 5, is this due to only household air pollution? Make it clear.

*We completely revised this to include the most recent evidence available and to add a reference to HAP and to the relevant year (2017). The relevant sentences now read as follows:*

*“Cooking with solid biomass such as wood and cow dung is a major source of household air pollution, which in turn is of one of the most important risks for global health, especially for women and children. Annually, millions of people die prematurely, with estimates for the years 2016 and 2017 ranging between 1.6 million [3] and 3.8 million [4].*

Line 15, …Fuel stacking ….. mention that it means multiple fuel use.

*The revised sentence reads as follows: “Multiple fuel use (so-called ‘fuel stacking’) is a widespread phenomenon and may persist over a long time.”*

- Line 25, …. Indeed, our interviews demonstrate…… is this interview prior to your study? with the study subjects? And did you include health information based on this interview result? I expect you to first introduce what your study is about.

*We had included this preview of some of our results in the introduction to motivate the reader by showing the relevance of health information. Yet, we agree that this is not really a good place to show these results. In the new version of the text, we deleted them from the introduction and, instead, added two paragraphs where we elaborated on the guiding research question, and the structure of the paper.*

*The findings from the interviews are now all included in the results part of the paper.*

- Line 31, …some general information ….. Did you also give this general information to the treatment group? Make it clear. This is where your reader should know the difference between the treatment and control groups.

*We clarified by revising this sentence as follows: “We randomly assigned health information to one part of the respondents and general, non-health-related information on LPG to the other part.”*

- Lines 32-35, ….. Make your hypothesis very clear. What have you hypothesised regarding the effect of the treatment on the outcome variables? It is not stated plainly, especially for the first variable.

*We revised and complemented the corresponding paragraph of the introduction. The key sentences now read: “This paper now examines the effect of health messaging in a context where the problem of high upfront cost is already taken care of through the PMUY. We expect that under these new conditions, the effect of health messaging on WTP and LPG consumption may be substantial.”*

- Number your sections, see general comment. As it appears now, it is difficult to make cross-referencing.

*We are constrained to follow the journal’s formatting rules in this matter.*

- Lines 52-55, …..majority of households underestimate the severity of the risks but consider information demonstrating severity salient .......Isn't this counter intuitive given you already said that "majority of households recognize that there are some health hazards related to indoor air pollution"? consider revising.

*When referring to “some” health hazards respondents usually mean transient irritations of eyes and throat, but nothing to really worry about. The really problematic consequences of traditional cooking are usually not known to them before our intervention and considered as very salient. We revised the corresponding sentence to clarify this point: “When confronted with information about the fact that the adverse effects of household air pollution go way beyond transient irritations of eyes and throat, but substantially increase the risk of several deadly illnesses, they consider this information as highly salient.”*

- Line 61…Mixed findings ….. Your review does not show that the findings in the literature are mixed. I cannot see a study that find that health information increases WTP for clean cooking solutions or adoption of improved stoves.

*Indeed the evidence for WTP and clean cookstoves is overwhelmingly negative. The available positive evidence we mention refers only to other dependent variables in response to HAP-related health messaging (e.g., cooking outside instead of inside the house). We have completely reformulated the entire subsection to clarify these differences.*

- Lines 64-68, repeated sentence

*The sentence is deleted.*

- Line 78, …..Tibet….where is it? China?

*Checking the reference revealed that the research was actually conducted in Myanmar. We corrected this error.*

- Line 86, ….does not provide consistent evidence…. What do you mean by this? information did not matter?

*The messages did increase willingness to pay only in one out of multiple settings. We clarified accordingly.*

- Line 89, mention these studies (RCTs)

*Since this section has been completely reformulated, the sentence does not exist anymore in its original form. When reformulating, we have made sure to cite the studies wherever they are first mentioned.*

- Line 134, did not you already mention on line 131 that load can arranged to buy the first cylinder? / and here free of charge/?

*We have attempted to clarify this point further by emphasizing that no repayment is required if there is no further purchase of LPG:*

*“Since no repayment is required if no further refills are not purchased, households generally consider the initial uptake of the new technology as free of charge.”*

*Furthermore, we now mention in the previous sentence that the loan is interest-free.*

- Line 146, I think, this is what you have done and will contribute to the literature. I suggest that you should elaborate this in the introduction.

*We now elaborate on our contribution – to test the effect of health messaging under the new financial conditions – in a new paragraph in the introduction.*

- Line 167, x includes …….revise/rewrite this sentence

*We revised this sentence, trying to clarify.*

- Lines 246-247…….It would be nice it you include a map of your study area.

*We now include a map showing Bikaner district (the study area) within the state of Rajasthan in S2 Appendix 2, which describes the sampling procedure. The text in the appendix is adjusted accordingly.*

- Line 260 …..electricity is more widespread …….consider revising/rephrasing it.

*Revised: “…access to electricity is higher in Bikaner than….”*

- Lines 263-267, sex ratio is over discussed. If you feel it helps to read your paper, write it in a footnote. The discussion diverts attention to a different topic.

*We substantially shortened the discussion of the sex ratio in Bikaner.*

- Line 282, your respondents are not in the households´ decision-making position, and therefore, your discussion should be streamlined taking this into account. I can see that you try to caution the reader, but you still appear to make strong conclusions. I strongly advise you to tone down your statements related to female-male treatment effects. See major comment 1.

*We revised our discussion substantially to address this issue. For details please see answer to major comment 1.*

- Line 347, the way you illustrate the diseases (presentation of pictures) per se may affect respondents’ mindsets and hence decisions. I think you should elaborate that the pictures are not disturbing or very sensitive to have such effects.

*We now state: “To illustrate the verbal information, the enumerators carried along colored plasticized picture cards (size A4). We selected images that would be as neutral as possible while visually clarifying the spoken text.”*

- Line 548, First indicate what each column in the table shows and then proceed to the explanations to make your table more readable.

*We revised the beginning of the subsection “Multivariate regression analysis for WTP” to follow this advice.*

- Table 2, column 3 and 4….it is not clear why the number of observations has decreased.

*For the variables added in columns 3 and 4, complete data is not available for the full sample, resulting in a smaller number of observations. We added this explanations to the notes of all tables where this is relevant.*

Line 581, …land ….. is this land ownership regardless of size?

*Yes. We clarified this now in the manuscript.*

- Line 615 …info…. write information in full

*It now reads: “Effect of health messaging on voucher use among voucher owners”*

- Line 706-707….I am sceptical regarding your calculation here. Intuitively, the demand for LPG (probability of voucher use) should not linearly increase from 4 to 10% when discount increases from 20 to 50 INR. This is because households will not keep linearly substituting other consumptions to LPG as they will experience satiation from increasing consumption of LPG (Marginal utility of LPG consumption would fall). At least, that is what your theoretical model implies. You cannot establish the equivalence, and if you do, you should make note of this.

*The numbers indeed correspond to the estimated average effect of price reductions when using a linear probability model (LPM) which constraints the impact to a linear effect. We agree that a linear model does not adequately reflect the probability distribution of the purchasing decision and that a probit model hence better corresponds to our theoretical model.*

*In the revised version, we discuss this issue in the manuscript and estimate a probit model with the same variables. We present the outcomes of this analysis in terms of the predicted margins of the treatment at different discount values in Fig 3 in S5 Appendix 5, and discuss them in the main text. The results suggest that, starting from the regulated price of LPG at the time of the field study, a substantially higher price reduction is now necessary to achieve the same effect as health messaging.*

*Interestingly, this suggests that, at least initially, the relative effectiveness of the health messaging will be even greater than suggested by our earlier wording.*

- Line 744-748….repeated sentence

*We deleted the repeated sentence.*

- Line 788…. Why is it particularly high in India?

*We now provide information to underline this statement. The revised sentence reads as follows: “With an estimated 846 million people being exposed to household air pollution, in India, the health burden from household air pollution is particularly high.”*

- Line 828…..important for what?

*This statement is not part of the conclusions anymore as we have substantially shortened and toned-down the discussion of potential gender effects.*

Reviewer #2: This paper explores the effect of providing health-related information on the choice of energy source for cooking. It uses an experimental design and compares WTP and actual LPG use as outcomes of interest that may (and did) vary between treatment and control groups. This is highly relevant both in terms of making the energy sector more efficient and mitigating avoidable premature deaths. To the extent that it touches big themes (energy and health), I would like to see the paper motivated in a stronger way by linking these themes to the sustainable development goals.

*Thank you very much for this suggestion, which we are happy to endorse. We now strengthened the motivation of the paper by adding a new first paragraph in the introduction. It mentions not only the health impact, but also the environmental and social impact of traditional biomass and based on this names a range of SDGs on which progress can be advanced by promoting access to clean residential energy. We also refer to SDG 7 in the abstract now, and mention the relevance of the topic for the SDGs in the first sentence of the conclusions.*

Regarding the information in the first paragraph of the introduction, if global premature death associated with health problems of using biomass cooking is 4 million, that of India’s being 1 million sounds too much. I didn’t do fact check but it doesn’t sound right: Either the former has to be bigger of the latter smaller, or the figures were observed at different times.

*Based on your comment, we have verified the numbers once more, which gave us the opportunity for some updating. In essence, however, these numbers remain the same: when using the same sources and years, the estimates one finds for India are more than 25% of the world’s total. The computational methods for the estimation significantly vary by sources, however, so that it is crucial to pick the same source for both. The most recent numbers for India are now from the State of global air report and refer to 2017, which estimates a death toll 482000 people. The same publication comes to a worldwide estimate of 1.6 million, however, which is also considerable less than the 3.8 million estimated by the WHO. We now provide the full range to be sure.*

The information in paragraph 4 (of the introduction) comes from the survey, but it is being used to justify why information intervention may induce change in behavior. I think the introduction can be structured in a better way. At this point, readers would expect to see the gap, research questions, hypotheses, contribution of the study etc.

*We substantially revised the introduction to follow this advice. The information coming from the survey is not part of the introduction anymore, but is placed in the results section instead. In addition, we added a new paragraph to elaborate on the guiding research question/expectation, and the contribution of the paper.*

Regarding the treatment, I don’t know how a poor person who can hardly afford it will change behavior and buy more only because they have enough information. Where will they get the money from? In the case of loan (line 130, page 4), they may also be evading repayment by not refilling. I think the central issue is not information but economics (which the authors highlight in different parts of the paper, e.g., lines 113-15).

*We agree that financial constraints will remain a major barrier to sustained LPG use and we highlight this issue in the paper. At the same time, with PMUY, the major initial investment is taken care of. We believe that this makes a huge difference. Indeed, prior surveys suggested these upfront costs constituted the main barrier for poor households’ energy transition. In order to buy refills, only smaller amounts are needed, and while they are still considerable for poor households, they can be made available through a change in consumption priorities in most cases. The purchase may still appear costly, but not completely out of range. We hope that the revised sub-sections “No consistent evidence for the effect of health messaging”, “Insufficient evidence in the LPG context” and “New situation as upfront costs are covered” clarify this point.*

*More generally, we believe that to guide policies to promote access to LPG for poor communities, it is important to improve our understanding on the relationship between the effect of information and the effect of price reductions. We now discuss this relationship in even more detail, moving beyond the linear estimation model (see S5 Appendix 5). The analysis shows that the health information we delivered has the same effect on voucher use as a price reduction in the range of 50-140 INR starting from the current price of 480 INR (dependent on estimates and treatment groups). As we show in the final section of the results part in the main text itself, the respective policy measures, i.e., health information and price reductions, can also usefully complement each other.*

I don’t see the relevance of the information provided in the paragraph that starts on line 89.

*The whole section has been reorganized, and irrelevant information has been deleted.*

The next paragraph (lie 94) reads: “Willingness to pay for improved…”. The question that comes to mind is where?

*Due to the reorganization of the whole section, this sentence does not appear anymore in the same form. However, we now systematically try to provide the information on regions or countries covered by the studies we cite in this section.*

The results show that health awareness increased from 13% to 48%. I think 48% is too low and it has implications on the reliability of the design. As it is an experiment, the participants should understand the protocol properly. As much as the authors see the 48% as evidence for improved health awareness, a case can be made about the 52% being evidence for low internal validity of the experiment. If every participant understood the message, it would be reflected in a sweeping percentage being health aware.

*Thank you for highlighting this point as it is indeed important to avoid misunderstandings here. In this context, we would like to highlight several points:*

*1. While the awareness of severe health risks increased from 13% to 48%, the share of individuals who are aware of some health risks (i.e. including those who believe there are health risks, but not very severe ones) increased to 94%. The section on the information channel now discusses the difference between both measures. Furthermore, we added the following sentence to make things explicit: “The total share of individuals who reported to be aware of health risks (serious or less serious) increased to 94\%.”*

*2. Similarly, in response to our post-intervention question whether LPG is better / similar / worse for health than the so-called chulha (traditional cookstove) (see Q1 in post-intervention questionnaire in S4 Appendix 4), 81% in the treatment group stated that LPG is better (vs. 57% in the control group).*

*3. In our view, the fact that a large share of respondents in the treatment group still responded that they were not aware of severe health effects does not necessarily mean that they did not understand the message. In particular, even if a message is understood, it may not necessarily be believed. This may be plausible here as for many people, this kind of information was something they had never heard about before. Even our enumerators (students at the Agricultural University of Bikaner) first had to be convinced that the information comes from reliable sources such as the WHO. Before the study, they did not know about the severe health effects either and were extremely surprised about this issue.*

When it comes to behavior elicitation, knowledge is important but experience may be vital. The authors could have asked if respondents had any serious health problems recently.

*In general, the health problems associated by household air pollution are very widespread among households using solid biomass as primary cooking fuel like our respondents and also in the state of Rajasthan more generally, as for instance the following study shows:*

https://www.researchgate.net/publication/327594267_The_burden_of_chronic_respiratory_diseases_and_their_heterogeneity_across_the_states_of_India_the_Global_Burden_of_Disease_Study_1990-2016_India_State-Level_Disease_Burden_Initiative_CRD_Collaborators

*Given how widespread these diseases are, we assume that experience of such diseases is not something that leads to a major differentiation between households.*

*To what extent people associate these hazards with traditional cooking practices is a different question. The association between the two is affected by our health-messaging intervention. In our post-intervention questionnaire, we do ask about the respondents’ experience of related health problems, but we do not consider that it would be useful to use the responses as a proxy of experience. Rather, they show the difference between treatment and control group in how well they link such diseases to HAP.*

*Indeed, only 22.5% of the control group state that they experienced severe health hazards related to cooking on the traditional stove. In the treatment group, however, the share of respondents that reports severe health hazards and relates them to cooking with traditional biomass is almost 58%.*

*We have now included the information on the questionnaire including these health-related questions in S4 Appendix 4, and refer to it in the text.*

It is possible that availability bias may cause differences in WTP.

*In the same village, the availability of LPG does not vary across experimental groups.*

Justification for the use of linear probability model (not probit/logit) is not strong enough. Yes, it is easier for interpretation but it would be better to explicitly state the gains and loses of making this choice.

*For most of our analysis, there is not much to gain or to lose, as the results are very similar. For the reader to be able to verify that, the revised version now provides the dataset and the code to run either the LPG or a probit model as supporting information in S6 Dataset. In the manuscript we added a clear reference to the code for running the probit models.*

*Furthermore, some probit results are now presented in S5 Appendix 5 for the relationship between price reductions and health messaging as alternative policy interventions and mentioned in the main text at the end of the section “Joint effect of health messaging on voucher use”. Here indeed, the non-linear presentation comes closer to the theoretical model, and its graphical presentation provides some interesting complementary insights.*

On writing: instead of saying “our study doesn’t lend itself…” (line 37, page 2), it is clearer to be direct and say “our study does not measure…). This is just an example, and the paper can be written more succinctly and precisely.

*We have followed this advice and now write explicitly: “In our study we did not measure any direct health outcomes.” Beyond this specific sentence, we have done our best to make the paper more concise and precise as suggested.*

Repetitions: Lines 64 and 67; paragraph on line 741.

*Thank you for noting this, we appreciate it. The repetitions mentioned have now been removed.*

Why are the number of households 550 but the number of individuals in the study 554?

*Thank you for noting the inconsistency. The revised version contains the correct number of individuals (= identical to number of households), which is 554. (550 households were initially foreseen based on our power calculations. Eventually, some replacement households were included given that not everyone who agreed to take the survey also answered all relevant questions and participated in the LPG experiment. 539 eventually completed the WTP exercise.)*

6. PLOS authors have the option to publish the peer review history of their article ([what does this mean?](https://journals.plos.org/plosone/s/editorial-and-peer-review-process#loc-peer-review-history)). If published, this will include your full peer review and any attached files.


If you choose “no”, your identity will remain anonymous but your review may still be made public.


**Do you want your identity to be public for this peer review?** For information about this choice, including consent withdrawal, please see our Privacy Policy.

Reviewer #1: No

Reviewer #2: No


[NOTE: If reviewer comments were submitted as an attachment file, they will be attached to this email and accessible via the submission site. Please log into your account, locate the manuscript record, and check for the action link "View Attachments". If this link does not appear, there are no attachment files to be viewed.]


While revising your submission, please upload your figure files to the Preflight Analysis and Conversion Engine (PACE) digital diagnostic tool,. https://pacev2.apexcovantage.com. PACE helps ensure that figures meet PLOS requirements. To use PACE, you must first register as a user. Registration is free. Then, login and navigate to the UPLOAD tab, where you will find detailed instructions on how to use the tool. If you encounter any issues or have any questions when using PACE, please email us at figures@plos.org. Please note that Supporting Information files do not need this step.
